# Determinants of Inter-Individual Variability in Corticomotor Excitability Induced by Paired Associative Stimulation

**DOI:** 10.3389/fnins.2019.00841

**Published:** 2019-08-14

**Authors:** Lora Minkova, Jessica Peter, Ahmed Abdulkadir, Lena V. Schumacher, Christoph P. Kaller, Christoph Nissen, Stefan Klöppel, Jacob Lahr

**Affiliations:** ^1^University Hospital of Old Age Psychiatry and Psychotherapy, University of Bern, Bern, Switzerland; ^2^Department of Psychiatry and Psychotherapy, Medical Center – University of Freiburg, Faculty of Medicine, University of Freiburg, Freiburg, Germany; ^3^Freiburg Brain Imaging, Medical Center – University of Freiburg, Freiburg, Germany; ^4^Department of Medical Psychology and Medical Sociology, Faculty of Medicine, University of Freiburg, Freiburg, Germany; ^5^Department of Neuroradiology, Medical Center – Faculty of Medicine, University of Freiburg, Freiburg, Germany; ^6^University Hospital of Psychiatry and Psychotherapy, University Psychiatric Services, University of Bern, Bern, Switzerland; ^7^Department of Neurology, University Hospital Bern, University of Bern, Bern, Switzerland; ^8^Center for Geriatrics and Gerontology Freiburg, Medical Center – Faculty of Medicine, University of Freiburg, Freiburg, Germany

**Keywords:** TMS, paired associative stimulation, resting-state fMRI, sensorimotor network, DTI, corticospinal tract, random forest

## Abstract

Transcranial magnetic stimulation (TMS) is a well-established tool in probing cortical plasticity *in vivo*. Changes in corticomotor excitability can be induced using paired associative stimulation (PAS) protocol, in which TMS over the primary motor cortex is conditioned with an electrical peripheral nerve stimulation of the contralateral hand. PAS with an inter-stimulus interval of 25 ms induces long-term potentiation (LTP)-like effects in cortical excitability. However, the response to a PAS protocol tends to vary substantially across individuals. In this study, we used univariate and multivariate data-driven methods to investigate various previously proposed determinants of inter-individual variability in PAS efficacy, such as demographic, cognitive, clinical, neurophysiological, and neuroimaging measures. Forty-one right-handed participants, comprising 22 patients with amnestic mild cognitive impairment (MCI) and 19 healthy controls (HC), underwent the PAS protocol. Prior to stimulation, demographic, genetic, clinical, as well as structural and resting-state functional MRI data were acquired. The two groups did not differ in any of the variables, except by global cognitive status. Univariate analysis showed that only 61% of all participants were classified as PAS responders, irrespective of group membership. Higher PAS response was associated with lower TMS intensity and with higher resting-state connectivity within the sensorimotor network, but only in responders, as opposed to non-responders. We also found an overall positive correlation between PAS response and structural connectivity within the corticospinal tract, which did not differ between groups. A multivariate random forest (RF) model identified age, gender, education, IQ, global cognitive status, sleep quality, alertness, TMS intensity, genetic factors, and neuroimaging measures (functional and structural connectivity, gray matter (GM) volume, and cortical thickness as poor predictors of PAS response. The model resulted in low accuracy of the RF classifier (58%; 95% CI: 42 − 74%), with a higher relative importance of brain connectivity measures compared to the other variables. We conclude that PAS variability in our sample was not well explained by factors known to influence PAS efficacy, emphasizing the need for future replication studies.

## Introduction

Transcranial magnetic stimulation (TMS) is a well-established non-invasive brain stimulation tool that can be used to probe cortical plasticity. Changes in corticomotor excitability can be induced using a paired associative stimulation (PAS; Stefan et al., 2000). This involves the repeated application of an electrical peripheral nerve stimulus (e.g., median nerve stimulation; MNS) paired with a single-pulse TMS to the primary motor cortex. The TMS leads to a contralateral muscle contraction that can be measured in the form of a motor evoked potential (MEP). PAS is related to Hebbian principle of activity-dependent long-term modification of synaptic plasticity (Hebb, 1949). Depending on the inter-stimulus intervals and stimulation duration, PAS may induce either long-term potentiation (LTP)-like or long-term depression (LTD)-like effects (Ziemann et al., 2008). Such shifts in corticomotor excitability are quantified by topographically specific changes in the MEP amplitudes.

The PAS protocol consists of a short pre-measurement period (i.e., baseline MEP), followed by the PAS intervention, and finally a post-measurement period to evaluate stimulation effects. PAS-induced LTP-like effects are associated with increased MEP amplitudes following stimulation. However, research shows that the PAS response is not always robustly elicited but is rather affected by considerable inter- and intra-individual variability (Müller-Dahlhaus et al., 2008; Ridding and Ziemann, 2010; Karabanov et al., 2016). For instance, a number of different studies have shown that PAS targeting the primary motor cortex elicited the expected effect in only 60% or less of all participants (for a review, see Karabanov et al., 2016). Due to this variability, the division into responders and non-responders has been used in previous works by applying a dichotomous cut-off (Müller-Dahlhaus et al., 2008; List et al., 2013b; López-Alonso et al., 2014; Klöppel et al., 2015; Lahr et al., 2016b). More specifically, the grand average of the post-stimulation sessions is calculated, normalized to the mean MEP at baseline. Participants are then divided into PAS responders (with post/baseline ratios above 1) and PAS non-responders (with post/baseline ratios equal to or below 1).

To date, fluctuations in post-stimulation effects among participants are poorly-understood. PAS-induced LTP-like effects have been reported to decrease with age (Müller-Dahlhaus et al., 2008) as well as in clinical populations, such as Alzheimer’s patients (Battaglia et al., 2007) and Parkinson’s patients (Morgante et al., 2006), among others. Other potential sources of intra-and inter-individual variability in responses to PAS include circadian fluctuations and time of day (Sale et al., 2007; López-Alonso et al., 2014), alertness (Kamke et al., 2012), attentional state (Stefan et al., 2004), sleep (Kuhn et al., 2016), stimulation intensity (Müller-Dahlhaus et al., 2008), as well as genetic traits (Missitzi et al., 2011), such as brain-derived neurotrophic factor (BDNF) polymorphism (Cheeran et al., 2008; Fried et al., 2017) and possibly also Apolipoprotein E (APOE) genotype (Peña-Gomez et al., 2012; Lahr et al., 2016b). Additionally, neuroanatomical determinants, such as cortical thickness (Conde et al., 2012; List et al., 2013b) and microstructural properties of white matter (WM) (Klöppel et al., 2008; List et al., 2013a), seem to influence cortical excitability, as well. Previously, it has been shown that resting-state functional connectivity patterns measured prior to repetitive TMS intervention in depression may also predict individual therapeutic response (Downar et al., 2014; Salomons et al., 2014).

All the various potential determinants of inter-individual variability in PAS efficiency have been investigated in isolation by different studies but have not been replicated systematically. Here, we propose a multivariate explorative approach to investigate to what extent PAS response rate can be predicted using different factors of variability, including demographic variables and factors (e.g., age, gender, education, IQ), genetic characteristics (e.g., BNDF, APOE), neuroanatomical measures (e.g., cortical thickness, structural and functional connectivity patterns), neurophysiological qualities (e.g., sleep quality, attention, alertness), and neuropsychological variables (cognitive status, depression). For this purpose, we used a Random Forest (RF) classifier, an ensemble machine learning algorithm, which consists of a collection of decision trees trained with different subsets of the original data (Breiman, 2001). Among the advantages of RF is that it is robust to noise, is invariant to the scaling of features, can handle high-dimensional, redundant data and can be used for ranking the importance of predictors by randomly permuting the values of each predictor at a time and estimating the decrease in prediction accuracy. The aim of our study is twofold. First, we hypothesize that a combination of different factors would be best suited to predict the efficiency of the PAS outcome, and, second, we aim to assess the hierarchical importance of these determinants of PAS variability, which could be used to inform future studies focusing on TMS-induced plasticity.

## Materials and Methods

### Participants

A total of 48 participants were included in the original study (Lahr et al., 2016b). Among them, 24 were patients with amnestic MCI and 24 were age-, sex- and education-matched healthy controls (HC). MCI were diagnosed as being amnestic if memory function was below 1.5 SD on verbal delayed recall (Petersen et al., 1999; Albert et al., 2011). One participant with Beck Depression Inventory (BDI-II; Beck et al., 1996) score of ≥13 and Geriatric Depression Scale (GDS; Yesavage and Sheikh, 1986) score ≥5 was excluded from the study, according to the cut-off score for a minor depressive syndrome. Further three participants were excluded due to corrupt or missing MRI scans, as well as three participants were excluded due to left-handedness, which was assessed using the Edinburgh Handedness Inventory (EHI; Oldfield, 1971). Further exclusion criteria included any history of severe neurological, psychiatric or other diseases, smoking, or any history of substance abuse. Thus, the final study sample comprised 41 participants (19 MCI and 22 HC). Patients were recruited from the Center for Geriatric Medicine and Gerontology of the Medical Center – University of Freiburg, Germany, while controls were recruited via newspaper advertisements and handouts circulated in Freiburg. The study was approved by the Ethics Commission of the Medical Center – University of Freiburg (Approval #227/12) and written informed consent was obtained from all participants prior to participation according to the Declaration of Helsinki.

### Study Procedure

Each participant took part in the study on two consecutive days. Prior to testing, all participants were asked to complete the questionnaire of handedness (EHI; Oldfield, 1971), the Pittsburgh Sleep Quality Index (PSQI; Buysse et al., 1989) to assess sleep quality over the 4 weeks prior to testing, as well as BDI and GDS to assess depressive symptoms. The total BDI and GDS depression scores were transformed into z-scores using the sample statistics and combined into one single composite score (i.e., the average of the two scores) for subsequent analyses. On the first study day, participants completed a neuropsychological battery including diverse short tests assessing executive functioning, verbal and non-verbal learning, episodic memory, and visuo-constructive abilities, as reported elsewhere (Lahr et al., 2016b; Peter et al., 2016, 2018a,b). Global cognitive functioning was evaluated using the Montreal Cognitive Assessment (MoCA; Nasreddine et al., 2005). Structural and functional MRI scans were also acquired during the first day of testing (for more detail, see sections “MRI Data Acquisition” to “Diffusion MRI” below). On the following day, TMS was performed in the afternoon and sleep quality between day 1 and day 2 was assessed using the Sleep Questionnaire A (SFA; Görtelmeyer, 1985). Alertness and selective attention were evaluated using the WAF Perception and Attention Functions Battery (Sturm, 2006) as part of Vienna Test System^1^. Finally, blood samples were also collected from all participants in order to determine APOE allele ε4 genotype and BDNF Val66Met polymorphism.

### PAS Protocol

The stimulation protocol was based on a previously published and widely used PAS paradigm (Stefan et al., 2000), in which TMS over the left primary motor cortex was conditioned by electrical stimulation of the right hand.

We performed TMS using a magnetic stimulator (Magstim 200; Magstim; Whitland, United Kingdom) with a figure-of-eight coil. The coil was positioned tangentially above the left primary motor cortex, with the handle pointing backward and rotated approximately 45° away from the midsagittal plane. The stimulation hotspot was defined as the optimal coil position to elicit motor responses in the contralateral abductor pollicis brevis (APB) muscle at suprathreshold stimulator intensity. The strength of the muscle contraction was recorded as motor evoked potentials (MEPs), the amplitude of which reflects cortical excitability from the targeted primary motor cortex. The stimulator intensity was adjusted in order to evoke a peak-to-peak MEP amplitude of 1 mV. MEPs were monitored online and amplified, bandpass-filtered (lowpass-filter: 8 kHz, time constant: 30 ms, corresponding to a cut-off frequency of 5.3 Hz) and digitized with an analog-to-digital converter at a sampling rate of 2 kHz (micro1401, Cambridge Electronic Designs, United Kingdom). Coil position and orientation were monitored and captured using an optical navigation system (Localite GmbH, Sankt Augustin, Germany).

Conditioning stimuli represented single pulses of electrical stimulation through bipolar electrodes applied to the median nerve at the right wrist, using a constant current stimulator (Digitimer DS7; Welwyn Garden City, United Kingdom). Electrical stimulation intensity was set to 300% of the individual perception threshold. The conditioning stimulus preceded the magnetic cortical stimulus by a time interval of 25 ms, which has been shown to result in facilitation of the MEP responses (Stefan et al., 2000). A total of 180 paired stimuli were applied at an interval of 5 s.

The PAS protocol consisted of three different conditions: one pre-measurement as a baseline (PRE), the intervention condition (PAS), and three post-measurement conditions: immediately after PAS (post1), after 8 min (post2), and after 15 min (post3), respectively. During the PRE and POST conditions, 20 TMS pulses were applied at an interval of 6 s and with a variability of 20% in order to prevent systematic MEP variability due to expectation. To keep participants attentive, they were presented landscape images on a screen during the PRE and POST conditions. When the PAS intervention started, they were asked to mentally count blue balls appearing on a computer screen. Ball counting was meant to ensure that participants did not close their eyes or fall asleep during PAS, but the total number of balls counted was not included in subsequent statistical analyses.

Trials with pre-facilitated activity were discarded manually, affecting on average 6.3 ± 1.8 out of 80 trials per individual. Based on previous literature, the three post-sessions were averaged and divided by the baseline amplitude in order to compute a marker of potentiation (Müller-Dahlhaus et al., 2008; List et al., 2013a,b). Based on the recommendations by a previous multi-centric analysis (Lahr et al., 2016a), we used the logarithms of the MEP amplitude ratio (i.e., post/baseline quotient) for subsequent analyses to reduce the possibility that results are driven by few extreme MEP measurements. Furthermore, we divided participants into two categories: PAS responders (log of MEP-ratio above 0) and non-responders (log of MEP-ratio equal to or below 0).

### Electric Field Simulation

The distribution of the electric field strength (i.e., the vector norm of the electric field E) induced by TMS was computed in SimNIBS (Version 2.1.1)^2^, based on the finite element approach using individual head models derived from the structural T1 and T2 MR images (Windhoff et al., 2013; Thielscher et al., 2015). Following the approach by Antonenko et al. (2018), the middle layer of the cortex was estimated for each participant based on segmentation results of the Computational Anatomy Toolbox CAT12 r1355^3^. Then, the position of the maximum electric field strength within the middle cortex layer was calculated as the TMS hotspot for each individual separately.

### MRI Data Acquisition

Scanning was performed on a 3 Tesla Siemens MAGNETOM TrioTim Syngo MR B17 scanner (Siemens Medical Systems, Erlangen, Germany) with a 12-channel phase array head coil. A high-resolution whole-brain T1-weighted anatomical image was acquired for each participant using the following magnetization-prepared rapid acquisition gradient echo (MPRAGE) sequence parameters: TR = 2200 ms, TE = 2.15 ms, FA = 12°, FOV = 256 mm, matrix size of 256 × 256 × 176 mm, and slice thickness of 1.0 × 1.0 × 1.0 mm, without a slice gap. Additionally, whole-brain T2^*^-weighted functional resting-state scans oriented along the AC-PC line were acquired for all participants using the following gradient echo-planar imaging (EPI) sequence: TR = 2610 ms, TE = 30 ms, FA = 80°, FOV = 192 mm, matrix size = 192 × 192 × 151 mm, 42 axially oriented slices acquired in a descending order, slice thickness of 3.0 × 3.0 × 3.0 mm, without a slice gap, and bandwidth of 2056 Hz/px. Resting-state scans consisted of 201 volumes. Participants were instructed to relax and passively stare at a fixation cross on a monitor display, keeping their eyes open during data acquisition. Diffusion-weighted images (DWI) were also acquired for each participant with the following acquisition parameters: TR = 10 s, TE = 94 ms, number of diffusion gradient directions = 61 (*b* = 1000 s/mm^2^), one image without diffusion weighting (*b* = 0 s/mm^2^), FOV = 208 mm, matrix size = 208 × 208 × 138 mm, slice thickness = 2.0 × 2.0 × 2.0 mm, and number of slices = 69.

### Structural MRI

Raw T1-weighted scans were visually inspected to ensure proper data quality and the absence of brain pathology (e.g., stroke or subdural hematoma). One participant was excluded due to poor data quality. All images were preprocessed using SPM12 v.6685 (Statistical Parametric Mapping, Welcome Trust Centre for Neuroimaging^4^) and the CAT12 r1355 (see footnote 3), running on MATLAB R2015a (Mathworks, Natick, MA, United States). They were first segmented into gray matter (GM), WM, and cerebrospinal fluid (CSF) using the IXI550_MNI152 template and the tissue probability map based on the Unified Segmentation (Ashburner and Friston, 2005). The segmented images were used to create an improved anatomical scan for subsequent co-registration of the functional images. Using the DARTEL extension for high-dimensional registration approach (Ashburner, 2007), deformation parameters were extracted for normalization of the functional images. CAT12 was used for voxel-based morphometry (VBM) to calculate GM and total intracranial volumes as well as for surface-based morphometry (SBM) to estimate cortical thickness based on the project-based thickness method (Dahnke et al., 2013). Region-of-interest (ROI) was the left precentral gyrus (Brodmann area 4) based on the Desikan-Killiany atlas (Desikan et al., 2006), which corresponds to the primary motor cortex. Regional GM volume was corrected for total intracranial volume (TIV) to account for individual brain size.

### Functional MRI

Preprocessing and functional connectivity of the resting-state fMRI data were completed using the CONN Toolbox v.18a (Whitfield-Gabrieli and Nieto-Castanon, 2012) in conjunction with SPM12. The first ten volumes were removed prior to preprocessing to avoid T1 equilibration effects. Preprocessing steps then included: slice-timing correction, realignment, coregistration to the anatomical image, normalization to MNI space, outlier detection (ART-based scrubbing), and smoothing with a Gaussian kernel (6 mm FWHM). None of the participants was excluded due to excessive head movement (motion artifact threshold: translation >3 mm, rotation >1°). One participant was excluded due to incomplete scans. A component-based noise correction (aCompCor) strategy (Behzadi et al., 2007) was used to remove the confounding effects of WM and CSF (five components each). Motion parameters were also regressed out (12 regressors: 6 motion parameters + 6 first-order temporal derivatives). Finally, the time-series were linearly detrended and band-pass filtered (0.01–0.08 Hz) to reduce noise effects and low-frequency drift.

Functional connectivity analysis was then performed using a whole-brain seed-to-voxel approach, where individual correlation maps were generated by extracting the mean resting-state BOLD time-series from the seed and calculating the correlation coefficients with the BOLD time-series of all other voxels. To compute the functional connectivity of the sensorimotor network, the left precentral gyrus based on the Desikan-Killiany atlas (Desikan et al., 2006) was used as a seed. The network was also replicated by replacing the seed with each individual’s TMS hotspot region that resulted from the electric field simulation analysis. Bivariate correlation coefficients were calculated using the General Linear Model (GLM) and a Fisher’s transformation was applied in order to normalize the data. Second-level (group) analysis within the CONN toolbox was used to compute and visualize the seed-based sensorimotor connectivity network across all participants, with *p*-uncorrected value <0.001 before applying the False Discovery Rate (FDR) correction at the cluster level (*p*_*FDR*_ < 0.05). Connectivity strengths were then extracted for further statistical analysis.

### Diffusion MRI

The DWI data were processed using standard FLS v.6.0 pipelines (Smith et al., 2004). The raw images were first corrected for eddy current distortions. The no-gradient (B0) image was skull-stripped using the Brain Extraction Tool (BET). Diffusion tensor fitting was completed using DTIFIT and fractional anisotropy (FA) values were derived from the tensors. Prior to fiber-tracking, crossing fibers within each voxel of the brain were estimated with a Bayesian method implemented in BEDPOSTX (Behrens et al., 2007). Probabilistic tractography of the corticospinal tract (CST) was computed in PROBTRACKX (Behrens et al., 2007) using pre-selected ROIs as seeds and targets based on previous literature (Wakana et al., 2004; Zhang et al., 2010; Chenot et al., 2018). More specifically, the left precentral gyrus was defined as a seed and the cerebral peduncle as a target. The internal capsule and the pons were defined as inclusion (i.e., waypoint) masks. In contrast, exclusion masks included the midline to remove pathways crossing into the other hemisphere. A WM termination mask was also used to ensure tracts stopped at the gray/white matter interface, thus discarding pathways extending into gray matter, CSF or dura. ROIs were created using the FSL Montreal Neurological Institute template and the Johns Hopkins University WM Labels Atlas (Mori et al., 2005). Connectivity distributions were generated from the seed regions in native space. The number of streamlines per voxel was set to 5000. The resulting images were then warped into diffusion space using the FMRIB’s Linear Image Registration Tool (FLIRT) and overlaid onto the B0 image for quality control. Each participant’s *FA* values were extracted from the CST for further statistical analysis. The tractography pathways of all participants were registered to FMRIB58_FA standard MNI space and averaged for visualization purposes.

### Statistical Analysis and Machine Learning

Statistical analysis was completed using R version 3.5.2 (R Core Team, 2016). First, demographic, clinical, and imaging data were compared between PAS responders and non-responders. Previously, we found no significant differences between controls and MCI (Lahr et al., 2016b), but report results here for completeness. Data normality was assessed using the Shapiro–Wilk test. Univariate statistical analysis was conducted using ANOVA/ANCOVA or Mann–Whitney U tests for continuous variables, as appropriate. Kruskal–Wallis test was used for ordinal variables and Chi-square test for dichotomous variables. Our analysis focused on the PAS response rate (responders vs. non-responders), which is a dichotomized variable with less statistical power. Therefore, we also completed a correlation analysis (Spearman’s rank correlation coefficient) using the log-transformed MEP ratio between the averaged post-measurements and baseline as a dependent variable. Correlation coefficients were converted to z-scores and compared between responders and non-responders. In all univariate analyses, a *p*-value < 0.05 (two-tailed) was considered significant. Adjustment for multiple comparisons was performed using the Benjamini–Hochberg method (Benjamini and Hochberg, 1995), controlling for FDR. Of note, due to the high number of variables and the relatively small sample size, the univariate analysis is exploratory in nature and may be affected by false-negative results.

Multivariate data analysis was conducted using a RF classifier, implemented in the *randomForest* R package (Liaw and Wiener, 2002). RF is an ensemble machine learning algorithm, which consists of a collection of decision trees trained with different subsets of the original data (Breiman, 2001). A detailed description of the algorithm is provided elsewhere (Liaw and Wiener, 2002). Briefly, the algorithm draws n_*tree*_ bootstrap samples from the original data and grows a classification tree for each of the bootstrap samples by sampling the predictors randomly (m_*try*_) and choosing the best split among those variables. After a large number of trees are generated, each RF classifier casts a vote for the most popular class. At each bootstrap iteration, out-of-bag (OOB) predictions (i.e., predicting the data not in the bootstrap sample using the tree grown with the bootstrap sample) are aggregated. On average, each data point would be OBB around 36% of the times. An OBB estimate of error rate (i.e., misclassification rates) is computed representing the classifier’s strength and dependence. RF also provides a measure of the importance of the predictor variables by looking at how much prediction error increases when OBB data for the variable is permuted, while all others are left unchanged.

We set the optimal number of trees (n_*tree*_) to 500 and ran the model 10 times in order to choose the number of random variables used in each tree (mtry). We chose *m*_*try*_ = 6 for our model, where the OOB error rate showed to stabilize and reach a minimum. We assessed the accuracy of the RF model in classifying between PAS responders and non-responders (outcome variable) using the *caret* R package (Kuhn et al., 2019). The ROC curve for RF was created using the *ROCR* R package (Sing et al., 2005). The following predictive variables were included in the model: demographic (age, sex, education, and IQ), clinical (MCI vs. HC, composite depression score, and global cognitive status based on MoCA), neurophysiological (sleep quality, attention, and alertness), genetic (APOE and BDNF), and MRI measures (cortical thickness, GM volume, functional and structural connectivity). TMS intensity (i.e., percent of maximal stimulator output) was also included as a predictor in the model. The importance of each variable was assessed using the mean decrease of accuracy, representing how much removing each variable reduced the accuracy of the model, as well as the mean decrease in Gini impurity index used for the calculation of splits in trees. Loosely speaking, the higher the values of mean decrease in accuracy and decrease in Gini score, the higher the importance of the variable in the model.

## Results

A detailed description of the cohort’s demographic and clinical information is presented in Table 1.

**TABLE 1:** Sample characteristics.

	***N***	**Overall (*n* = 41)**	**HC (*n* = 22, 54%)**	**MCI (*n* = 19, 46%)**	**Responders (*n* = 25, 61%)**	**Non-responders (*n* = 16, 39%)**
**Demographic, cognitive and clinical data**						
Groups						
HC	22	22 (54%)	–	–	11 (50%)	11 (50%)
MCI	19	19 (46%)	–	–	14 (74%)	5 (26%)
Age (years)	41	70.2 (5.5)	69.5 (5.9)	71.5 (4.9)	70.3 (5.0)	69.9 (6.5)
Gender (males)	41	24 (58%)	14 (63%)	10 (53%)	15 (60%)	9 (57%)
Education (years)	41	13.0 (7–20)	14.5 (7–20)	13.0 (8–20)	13.0 (7–20)	13.5 (9–20)
MWT-B IQ	41	124 (97–143)	127 (97–143)	121 (97–136)	118 (97–143)	124 (100–136)
MoCA score	40	26 (17–30)	27 (22–30)	23 (17–29)	25 (17–30)	27 (19–30)
BDI-GDS z-score	41	−0.07 (0.90)	−0.14 (0.81)	0.16 (0.98)	0.02 (0.95)	−0.03 (0.83)
**Sleep, attention and alertness**						
SFA-SQ score	40	4.1 (1.9–5.2)	4.2 (1.9–5.0)	3.9 (2.1–5.2)	4.2 (2.1–5.2)	3.9 (1.9–4.9)
PSQI score	40	6 (2–16)	5 (2–14)	6 (2–16)	6 (2–16)	5 (3–9)
WAF (RT in ms)						
Alertness (intrinsic)	40	236 (66)	212 (50)	263 (84)	232 (72)	243 (57)
Alertness (phasic)	41	221 (88)	207 (70)	237 (110)	216 (98)	228 (75)
Selective attention	39	355 (138)	342 (110)	371 (171)	332 (123)	390 (143)
**Genetic traits**						
BDNF (Val66Met)	39	15 (38%)	8 (36%)	7 (41%)	10 (41%)	5 (33%)
APOE4 ε4 carriers	35	17 (49%)	7 (37%)	10 (63%)	12 (55%)	5 (39%)
**TMS data**						
PAS response (log)	41	0.05 (0.2)	0.02 (0.1)	0.07 (0.2)	0.2 (0.2)	−0.2 (0.1)
TMS intensity (%)	41	50 (35–82)	54 (38–82)	49 (35–72)	49 (35–82)	54 (38–65)
PAS responders	41	25 (61%)	11 (44%)	14 (56%)	−	−
**Imaging data**						
CT of M1	41	2.1 (0.1)	2.1 (0.2)	2.1 (0.1)	2.1 (0.1)	2.0 (0.2)
GMV of M1 (TIV_corr._)	41	0.1 (1.1)	0.2 (1.3)	0.1 (0.1)	0.2 (0.9)	0.5 (1.3)
FA of CST	41	0.5 (0.2–0.5)	0.5 (0.4–0.5)	0.5 (0.2–0.5)	0.5 (0.3–0.5)	0.4 (0.2–0.5)
FC of M1-S1	41	0.1 (0.1)	0.2 (0.1)	0.1 (0.1)	0.2 (0.1)	0.1 (0.1)

*Data are provided as mean (SD), median (IQR), or *n* (%). HC, healthy controls; MCI, mild cognitive impairment; MWT-B IQ, multiple-choice word intelligence test, version B; MoCA, Montreal Cognitive Assessment; BDI-GDS z-score, composite score of the beck depression inventory and the geriatric depression score; SFA-SQ, sleep questionnaire A – sleep quality; PSQI, Pittsburgh Sleep Quality Index; WAF, Perception and Attention Functions Battery; BDNF, brain derived neurotrophic factor; APOE4, apolipoprotein allele 4; PAS response, log-transformed MEP ratio between the averaged post-measurements and baseline; CT, cortical thickness; M1, primary motor cortex; S1, primary somatosensory cortex; GMV, gray matter volume; TIV, total intracranial volume; FA, fractional anisotropy; CST, corticospinal tract; FC, resting-state functional connectivity z-scores.*

### Demographic, Cognitive and Clinical Data

Of the 41 participants included in the study, 22 (54%) were HC and 19 (46%) were MCI. The two groups did not differ in age (*F*_(__1__,__39__)_ = 2.098, *p* = 0.156), gender (HC: 14 males, MCI: 10 males; *X*^2^_(__1__)_ = 0.156, *p* = 0.693), or education (Mann–Whitney *U* = 243, *p* = 0.378). No difference was also found between genetic factors such as the presence of APOE allele ε4 genotype (*X*^2^_(__1__)_ = 0.138, *p* = 0.241) or BDNF Val66mMet polymorphism (*X*^2^_(__1__)_ = 0, *p* = 1). As expected per definition, controls had significantly higher MoCA scores than MCI (*U* = 359, *p*_*FDR*_ < 0.001), even after adding age, gender, and education as covariates, but no difference was found for the IQ score (*U* = 258, *p* = 0.102). In terms of sleep, alertness, and attention, groups differed only in intrinsic alertness (*F*_(__1__,__38__)_ = 6.532, *p*_–uncorr._ = 0.014), but this effect did not survive the FDR correction.

### TMS Data

The PAS intervention led to an increase in MEP amplitude in only 61% of all participants included in this study. Responder rate (responders vs. non-responders) did not differ according to group (*X*^2^_(__1__)_ = 1.511, *p* = 0.219) or gender (*X*^2^_(__1__)_ = 0, *p* = 1). Using 4 × 2 repeated-measures ANOVA analysis (TIME: Baseline (PRE), Post1, Post2, Post3 and GROUP: HC, MCI), we found no significant effect for TIME (*F*_(__1__,__39__)_ = 0.154, *p* = 0.697) or for the TIME x GROUP interaction (*F*_(__3__,__117__)_ = 0.776, *p* = 0.510), even if only responders were included in the analysis (*F*_(__3__,__69__)_ = 2.34, *p* = 0.081).

**FIGURE 1:**
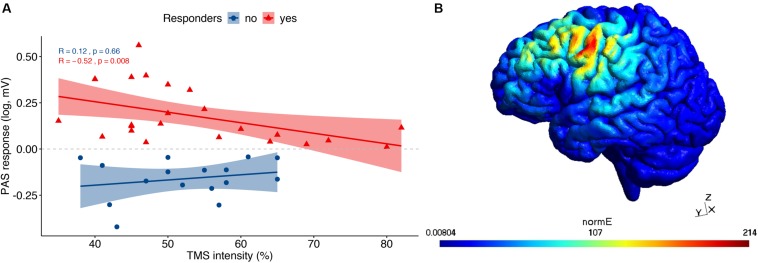
TMS results: **(A)** Correlational analysis between TMS intensity and PAS response, divided into responders and non-responders. **(B)** An exemplary TMS-induced field distribution from the simulation computed in SimNIBS.

The correlation analysis revealed a weak, non-significant negative association between PAS response (i.e., the logarithm of the MEP amplitude ratio) and age (*r*_*s*_ = −0.22, *p* = 0.17), which did not differ between HC and MCI, or between responders and non-responders. In terms of stimulation strength, TMS intensity was negatively correlated with PAS response (Figure 1A), but only in responders (*r*_*s*_ = −0.52, *p* = 0.008) as opposed to non-responders (*r*_*s*_ = 0.12, *p* = 0.66). However, no association was found between TMS intensity and PAS response when dividing the groups into HC (*r*_*s*_ = −0.029, *p* = 0.9) and MCI (*r*_*s*_ = −0.35, *p* = 0.051).

Using the SimNIBS toolbox, we visualized the location of the TMS hotspot to verify that the hotspot was within the motor cortex. An exemplary TMS-induced field distribution is illustrated in Figure 1B, while each individual’s coordinates in MNI space are provided in the Supplementary Table S1.

### Functional and Structural Data

**FIGURE 2:**
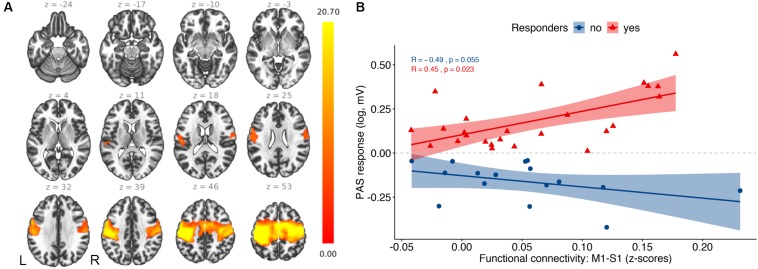
Resting-state fMRI results: **(A)** Average functional connectivity map of the sensorimotor network with the TMS hotspot as a seed (*p*_–_*_*FDR*_* < 0.05). The color bar represents *T*-values. **(B)** Correlation analysis between PAS response and functional connectivity between primary motor cortex (M1) and primary somatosensory cortex (S1).

The seed-based functional connectivity analysis of the resting-state fMRI group data revealed a consistent sensorimotor network (SMN; Figure 2A). More specifically, the SMN comprised one large cluster that overlapped with the following brain regions (Table 2): bilateral precentral gyrus, corresponding to the primary motor cortex (M1), bilateral postcentral gyrus, including the primary sensorimotor cortex (S1), supplementary motor area (SMA), bilateral superior parietal lobule (SPL), and bilateral supramarginal cortex (SMG). PAS response and functional connectivity of M1-S1 were positively correlated in responders (*r*_*s*_ = 0.45, *p* = 0.023) and negatively correlated at trend levels in non-responders (*r*_*s*_ = −0.49, *p* = 0.055) and correlation coefficients differed between the groups (z = 2.918, *p* = 0.001; Figure 2B). This effect was not observed when dividing the groups into HC (*r*_*s*_ = 0.3, *p* = 0.18) and MCI (*r*_*s*_ = −0.032, *p* = 0.9).

**TABLE 2:** Functional connectivity results: brain regions that positively correlated with the TMS hotspot seed.

**Anatomical labels**	**Cluster size (voxels)**	**MNI coordinates**			***T***	***p*_–FDR_**
				
		***x***	***y***	***z***		
Postcentral gyrus (S1) L	3708	−50	−22	52	20.70	<0.001
Precentral gyrus (M1) L	3548	−38	−10	62	19.36	<0.001
Precentral gyrus (M1) R	3413	24	−10	58	14.34	<0.001
Postcentral gyrus (S1) R	3065	52	−14	56	14.14	<0.001
Supplementary motor area (SMA)	1021	2	−6	52	16.46	<0.001
Superior Parietal Lobule (SPL) L	882	−38	−42	62	15.25	<0.001
Superior Parietal Lobule (SPL) R	650	24	−40	68	10.38	<0.001
Supramarginal gyrus (SMG) L	401	−60	−26	42	9.38	<0.001
Supramarginal gyrus (SMG) R	251	50	−26	44	7.66	<0.001

**FIGURE 3:**
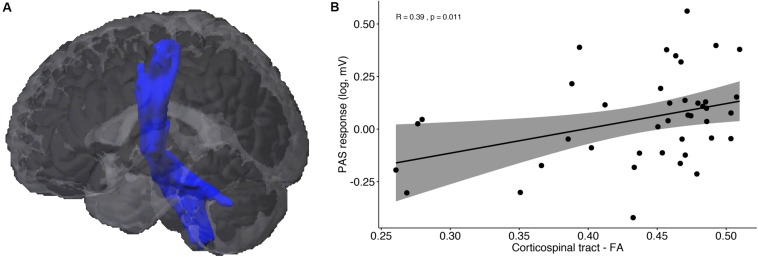
DTI results: **(A)** Probabilistic fiber tractography of the corticospinal tract (CST). **(B)** Correlation analysis between PAS response and average fractional anisotropy (FA) of the CST.

Figure 3A illustrates the corticospinal tract (CST), averaged across all participants, resulting from the probabilistic fiber tractography analysis. The weighted average *FA* values did not differ between MCI and HC (*U* = 162, *p* = 0.224), and showed only trend significance between responders and non-responders (*U* = 127, *p* = 0.053). The correlation analysis showed a significant positive correlation between *FA* values and PAS response across all participants (Figure 3B; *r*_*s*_ = 0.39, *p* = 0.011), but no difference in the correlation coefficients between responders and non-responders (z = 0.741, *p* = 0.229). In terms of cortical thickness and GM volume of the primary motor cortex, no significant differences between groups or associations with PAS response were found.

### Prediction of PAS Response Rate

**FIGURE 4:**
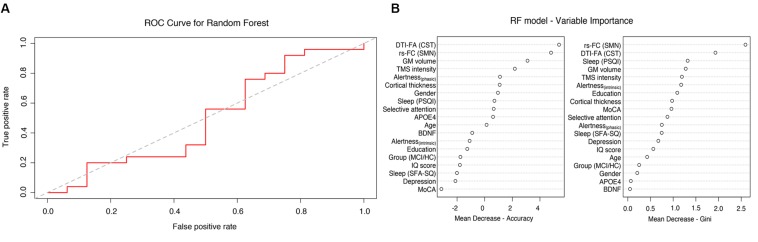
Random forest analysis: **(A)** Receiver operating characteristic (ROC) curves for the RF model with 19 features (AUC 0.49). **(B)** Importance of single variables.

A RF classifier was trained with *n*_*tree*_ = 500 and *m*_*try*_ = 6 using 19 different features in order to classify participants into two groups: PAS responders and non-responders. The RF classifier was not able to reliably predict the PAS response rate, showing a low estimated accuracy of 58% (95% CI: 42–72%). The results of the OBB estimations (i.e., confusion matrix) are shown in Table 3. We observed an estimated classification sensitivity [TP/(TP + FN)] of 65% and an estimated classification specificity [TN/(TN + FP)] of 50%, suggesting high susceptibility to large Type I error (false positives) of the model. The receiver operating characteristic (ROC) curve for the classifier is illustrated in Figure 4A. The area under the curve (AUC) was 0.49. The relative importance of variables is summarized in Figure 4B, showing that brain connectivity measures (i.e., microstructure of CST and functional connectivity of SMN) had the highest Gini impurity index. However, it should be noted that while RF can handle correlated variables well, multicollinearity may affect the relative importance of variables and should be interpreted with caution. The correlation matrix of all variables is provided in the Supplementary Figure S1.

**TABLE 3:** OBB estimation confusion matrix.

		**REFERENCE**	
		**PAS responders**	**PAS non- responders**
PREDICTION	PAS responders	TP = 20	FP = 5
	PAS non-responders	FN = 11	TN = 5

*TP, true positive; TN, true negative; FN, false negative; FP, false positive.*

## Discussion

Paired associative stimulation (PAS) is a well-established method to non-invasively probe cortical plasticity *in vivo*, but PAS effects tend to vary considerably among individuals. In this study, we addressed this issue by investigating the role of different factors that may affect PAS efficacy. In line with previous reports (Müller-Dahlhaus et al., 2008; López-Alonso et al., 2014; Lahr et al., 2016a), we found that only 61% of all participants included in our study showed the expected MEP facilitation as a function of the PAS intervention. More importantly, responder rate was independent of whether participants were HC or MCI. Using uni- and multivariate data analyses, we sought to determine if the observed high inter-individual variability could be predicted by factors that have previously been reported to influence PAS response, such as demographic, clinical, genetic, neurophysiological or neuroanatomical ones. We hypothesized that no single variable would be sufficient to predict the PAS outcome but expected that a combination of different determinants would have a synergetic effect on the predictability of the PAS response. Contrary to our expectations, our multivariate model could not sufficiently predict PAS response rate using these previously proposed determinants of PAS variability. To our knowledge, this is the first study to attempt predicting PAS efficacy using a multivariate classifier.

### Demographic, Clinical, and Genetic Factors

From the demographic data, age is considered a major factor that is known to influence LTP-like cortical plasticity and sensorimotor integration (for a review, see Bhandari et al., 2016). For instance, PAS-induced changes in MEP amplitude have previously been reported to be substantially smaller in elderly healthy individuals relative to young ones (Müller-Dahlhaus et al., 2008), while another study found only a trend toward a main effect of age, with young adults showing increases in MEP amplitude relative to older adults (Dickins et al., 2017). Here, we found only a trend for a negative association between age and PAS response, with no significant differences between HC and MCI, or between PAS responders and non-responders. However, it should be noted that the current study was not explicitly designed to investigate age-dependent effects on PAS-induced plasticity. Particularly, we included only older adults with a relatively narrow age range (60–77 years). While we agree that age should be regarded as an important confounding factor in TMS studies in general, we argue that PAS variability found in our study was not due to age differences. The same was also the case for other demographic determinants, such as sex, education, IQ, and global cognition.

The effects of clinical measures, including depression score and presence of cognitive impairment, were also considered in our analysis, motivated by previous findings. For instance, a recent study reported that depression may impair PAS-induced plasticity, with depressive patients showing lower PAS potentiation compared to HC (Noda et al., 2018). However, we found no correlation between depressive symptoms and PAS response, as well as no difference in depression scores between PAS responders relative to non-responders. This suggests that the inter-individual PAS variability observed in our sample could not be explained by depressive symptoms. Of note, depression symptoms were among the exclusion criteria in the current study in order to ensure that cognitive complaints in MCI were not due to depression. The initial goal of the study was to investigate whether PAS-induced plasticity differed between MCI and HC (Lahr et al., 2016b). Not only did we not confirm this hypothesis, but we also found that almost the same amount of HC were PAS non-responders, as observed in the MCI group. Since we found no main effect of group (except for MOCA) or interaction effects of group with any of the variables, we included the whole sample in the multivariate analysis. Limiting the analysis to controls only would have decreased statistical power without changing the conclusions of the study. Importantly, MCI is not a specific disease, but rather represents a “stage” along the aging continuum that does not necessarily need to progress to dementia. By definition, MCI participants present with mild cognitive deficits that do not impair their abilities to carry out normal daily activities.

In general, there is only limited evidence supporting the clinical application of PAS, especially at prodromal stages of neurodegenerative diseases (Ziemann et al., 2008). So far, impaired M1 plasticity has been reported in Parkinson’s disease (Morgante et al., 2006). Similarly, a previous study observed a PAS-induced increase in MEP amplitude in HC relative to patients with Parkinson’s disease and essential tremor (Lu et al., 2016). In the cognitive domain, only a few studies have focused on TMS-induced plasticity in Alzheimer’s disease (for a review, see Freitas et al., 2011), with some contradicting findings. More specifically, while most studies reported either no differences or decreased cortical excitability in AD (e.g., Battaglia et al., 2007), others suggested a short-term increase in post-intervention MEP amplitudes (Alagona et al., 2001). Interestingly, it has recently been suggested that a combination of different TMS paradigms may differentiate AD from frontotemporal dementia (Benussi et al., 2017).

The differences in findings among studies could be resulting from modulatory effects of potential pharmacological agents, as well as from the synergetic influence of genetic factors, such as APOE4 and BDNF. In particular, the impact of BDNF polymorphism on cortical excitability has been confirmed in mice (Fritsch et al., 2010) as well as in humans (for a review, see Chaieb et al., 2014). For instance, Kleim et al. (2006) showed in a TMS study that training-dependent facilitation of MEP amplitude was reduced in young healthy participants with a Val66Met polymorphism relative to those without the polymorphism. Moreover, Val/Val participants showed increased motor map areas of muscle representation, measured on T1-weighted images, relative to Val/Met and Met/Met participants, but this effect was only present after repeated training, suggesting that the physiological consequences of BDNF polymorphism may not manifest in the basal state but only occur in response to training-driven increases in neural activity, e.g., by reducing BDNF secretion in response to neuronal stimulation (Kleim et al., 2006). However, the small sample size (9 Val/Val, 11 Val/Met and 6 Met/Met participants) included in the study warrants some caution in interpreting these results. Here, we did not find a differential effect of BDNF polymorphism on PAS-induced plasticity but, possibly, the association between BDNF polymorphism and LTP-like facilitation may not manifest after a single PAS session.

### Neurophysiological and Neuroanatomical Factors

Following previous recommendations (Sale et al., 2007), the PAS experiment was completed in the afternoon for all participants. In this way, we aimed to avoid potential effects of circadian rhythms and time of day effects, thus providing a greater reproducibility between sessions. Furthermore, we evaluated sleep quality within the past 4 weeks as well as during the night prior to testing to ensure that PAS after-effects were not dependent on sleep. Previously, it has been shown that sleep deprivation leads to decreases in TMS-induced plasticity (Kuhn et al., 2016). In the present study, sleep quality did not significantly differ between groups and was not associated with PAS response.

With regard to controlling for attention and alertness during the TMS session, there is currently no consensus on the optimal approach. Here, we used a simple visual task to ensure that participants kept attentive and did not fall asleep during the session. It has previously been shown that a low visual load had no modulatory effects on PAS (Kamke et al., 2012). In addition, selective attention and alertness were evaluated on the day before the TMS session and no correlation was found with PAS response, which is in contrast to a similar study investigating TMS-induced plasticity in young adults, where we showed that higher LTP-like plasticity, in both motor and visual system, was associated with higher subjective alertness (Klöppel et al., 2015). While sleep, attention, and alertness are undoubtedly important factors to control for in brain stimulation interventions, here we found no significant associations with PAS after-effects, lending support to the idea that PAS variability in our study was caused by different factors.

Among all the determinants of PAS variability investigated in our study, the most promising ones seemed to be functional and structural connectivity measures. Neuroanatomical determinants, including cortical thickness, GM volume and microstructural properties of WM have previously been proposed to affect cortical excitability (Klöppel et al., 2008; Conde et al., 2012; List et al., 2013a,b). Our findings suggested that only the microstructure of the CST had a small, albeit significant, contribution to PAS efficacy. However, other studies, using tract-based spatial statistics (TBSS) of the corticospinal tract (CST) in healthy adults (Hübers et al., 2012) and in patients with Parkinson’s disease and essential tremor (Lu et al., 2016), showed that CST microstructure did not play a significant role in the generation of LTP-like plasticity. It is unclear whether these discrepancies are merely due to methodological differences among studies and, thus, warrants further examination. Considering that the CST is the major afferent pathway of the motor cortex, it is reasonable to expect that its anatomical integrity would be essential in defining the final motor output.

An advantage of functional connectivity measures over anatomical measures is their potential to provide useful insights into individual brain states as well as the effects of cortical excitability on neural processing. Although there is little understanding of the mechanisms underlying complex network organization and TMS-induced neuromodulation, available data highlight the utility of using task-based and resting-state fMRI to predict cortical excitability and TMS intervention outcomes (Fox et al., 2012, 2014; Cárdenas-Morales et al., 2014; Heba et al., 2017; Fiori et al., 2018; Ingemanson et al., 2019). For instance, a recent review on resting-state fMRI and treatment response in major depressive disorder reported that response to repetitive (rTMS) was consistently predicted by subcallosal cortex connectivity. Additionally, connectivity within default mode and cognitive control networks differed between treatment-resistant and treatment-sensitive patients (Dichter et al., 2015).

In our study, resting-state connectivity within the sensorimotor network was positively correlated with PAS-induced cortical plasticity, but only in responders relative to non-responders. In contrast, a previous study investigating intermittent theta-burst stimulation (iTBS)-induced increases in MEP amplitude found that resting-state connectivity did not predict iTBS after-effects (Cárdenas-Morales et al., 2014). However, they showed that task-dependent effective connectivity between left premotor areas and M1 prior to stimulation was predictive of post-intervention M1 excitability, implying that plasticity-related changes seem to depend on brain connectivity within the task-dependent network.

Of note, our study showed a correlational effect of SMN connectivity, but limited predictive value of PAS efficacy, as evident from our multivariate analysis. In order to evaluate causal effects of stimulation protocols on changes in functional connectivity, we propose that studies should acquire fMRI data both prior and following TMS interventions. Moreover, task-based fMRI designs might be better suited to investigate specific task-dependent changes in network connectivity as well as the short-term transfer of TMS-induced plasticity. An alternative approach to study the neuronal communication within the sensorimotor network is the use of bifocal, cortico-cortical PAS protocols, in which an impulse over the target area (e.g., M1) is followed by a second impulse over an interconnected target area (e.g., premotor areas) in an inter-stimulus interval consistent with the activation of short-latency connections between the two target areas (Rizzo et al., 2009; Arai et al., 2011; Buch et al., 2011). Recently, it was shown that this kind of modified PAS protocol cannot only induce cortical plasticity but also improve performance on a motor task involving the stimulated pathway (Fiori et al., 2018).

### Further Methodological Factors

An important consideration is whether the electrical field of the TMS indeed targeted the motor cortex with the intended direction and strength. In our study, we defined the stimulation hotspot as the optimal cortical location to elicit MEPs in the contralateral APB muscle. Furthermore, we used a neuronavigation system and each individual’s anatomical scan to register and track the coil position during the whole TMS session. In this way, we effectively controlled for motion effects since minimal movements away from the optimal stimulation region may lead to attenuation of the MEP amplitude. Additionally, using the SimNIBS software, we examined the distribution of the electric field strength and the coordinates of the stimulation hotspot for each participant separately in order to ensure that we indeed targeted the motor cortex.

It can be argued that defining the optimal TMS hotspot by using brain stimulation might not be optimal. Indeed, this approach has both its strengths and limitations. One shortcoming is that the search for the optimal hotspot might take longer in some participants than others, leading to an unanticipated bias. An alternative approach would be to define a neuroanatomical hotspot by first segmenting the individual’s T1 scan prior to the TMS session and then using an anatomical landmark, such as the left precentral gyrus. However, a limitation of this approach is that it does not take into account that the motor cortex consists of functionally and histologically distinct subregions and there is still no consensus which motor subregion should ideally be targeted (Bungert et al., 2017). In our study, we chose to functionally define the hotspot using the motor-evoked response and then inspect whether the coordinates of the TMS hotspot corresponded to the motor cortex.

Another methodological aspect is the choice of stimulation intensity. As generally recommended, we did not use a fixed intensity across all participants but, instead, defined it as the strength that evoked a peak-to-peak MEP amplitude of 1 mV, therefore ensuring that it was sufficient to induce the expected plasticity changes in each participant. Interestingly, our results suggest that higher TMS intensity does not lead to higher cortical excitability *per se*, emphasizing the importance of response-dose dependencies. Furthermore, TMS intensity can be influenced by (neuro-)anatomical features such as skull and cortical thickness, leading to individual differences in coil-to-cortex distance (McConnell et al., 2001; List et al., 2013b). To overcome this issue, we computed the distribution of the electric field strength (i.e., the vector norm of the electric field E) induced by TMS using the SimNIBS simulation approach, which takes into account neuroanatomical features such as CSF-skull boundaries and gyrification patterns, thus providing an anatomically more accurate modeling (Thielscher et al., 2015).

### Implications and Future Directions

Taken together, our study suggests that inter-individual variability in responsiveness to PAS was present even if variables known to influence cortical excitability were controlled for, highlighting the need for further replication studies. A major limitation of our study is that several of the variables had a relatively small range since the initial design of the study aimed to control for potential confounders. Therefore, it could be argued that the low predictive value of our multivariate model in terms of the inter-individual variability in PAS response is not surprising. Still, our findings have important implications, as we show empirically that low PAS responders rates are still present, even after controlling for potential confounding variables. Therefore, the underlying sources of variability in PAS efficacy are not well-understood and warrant further investigation. We put a special emphasis on the importance of avoiding publication bias by encouraging authors to publish negative results as well as to report non-responders in their analyses. Additionally, the generalizability of findings can be improved by optimizing sample size in order to ensure sufficient statistical power. Alternatively, future studies may refine their selected population by first evaluating individual state-dependent measures in order to homogenize the study sample. Modifications of existing protocols, instead of applying protocols in a “one-size-fits-all” fashion, may improve intervention outcomes (Karabanov et al., 2016). If PAS is to be used as a biomarker of cortical plasticity, a better mechanistic understanding of the variability in the responsiveness to PAS, as well as to other non-invasive brain stimulation protocols in general, is necessary. In line with previous recommendations (Karabanov et al., 2016), we emphasize that future studies should further focus on the application of state-informed open-loop (i.e., offline feedback) stimulation protocols (e.g., by using fMRI data to assess changes in brain states prior to and following stimulation), as well as the application of adaptive closed-loop (i.e., online feedback) approaches (e.g., by use of neurofeedback).

## Data Availability

The data that support the findings of this study are available from the corresponding author upon reasonable request.

## Ethics Statement

This study was approved by the Ethics Commission of the Medical Center – University of Freiburg (Approval #227/12) and written informed consent was obtained from all participants prior to the participation according to the Declaration of Helsinki.

## Author Contributions

LM acquired, analyzed, and interpreted the data, and drafted the manuscript. JP, SK, and JL designed the study, acquired, analyzed, and interpreted the data, and revised the manuscript. LS acquired the data and revised the manuscript. AA, CK, and CN interpreted the data and revised the manuscript. All authors read and approved the final manuscript.

## Conflict of Interest Statement

The authors declare that the research was conducted in the absence of any commercial or financial relationships that could be construed as a potential conflict of interest.

## Footnotes

1 https://www.schuhfried.at/test/WAF

2 https://simnibs.github.io/simnibs/build/html/index.html

3 http://www.neuro.uni-jena.de/cat/

4 http://www.fil.ion.ucl.ac.uk/spm
