# Supplementary material for: Determinants of Inter-Individual Variability in Corticomotor Excitability Induced by Paired Associative Stimulation
Source: Front Neurosci. 2019 Aug 14;13:841. doi: 10.3389/fnins.2019.00841 (PMC6702284; doi:10.3389/fnins.2019.00841)

**Table S1**. Individual coordinates of TMS hotspot seeds.

| **Participant ID** | **MNI Coordinates** | | |
| --- | --- | --- | --- |
|  | **x** | **y** | **z** |
| F0105 | -38 | -22 | 65 |
| F0194 | -51 | -6 | 43 |
| F0319 | -50 | -9 | 49 |
| F0623 | -46 | -14 | 46 |
| F0634 | -42 | -17 | 59 |
| F1366 | -46 | -8 | 55 |
| F1471 | -42 | -16 | 58 |
| F2255 | -53 | -11 | 45 |
| F2576 | -52 | -11 | 51 |
| F2634 | -42 | -17 | 61 |
| F2796 | -45 | -12 | 56 |
| F2861 | -31 | -22 | 69 |
| F2905 | -41 | -18 | 60 |
| F2985 | -51 | -12 | 42 |
| F3037 | -43 | -13 | 56 |
| F3488 | -34 | -24 | 68 |
| F3551 | -41 | -20 | 65 |
| F3865 | -42 | -12 | 63 |
| F4035 | -44 | -14 | 60 |
| F4046 | -36 | -20 | 68 |
| F5415 | -41 | -16 | 62 |
| F5702 | -39 | -20 | 61 |
| F5755 | -44 | -15 | 57 |
| F6177 | -36 | -22 | 65 |
| F6344 | -53 | -8 | 40 |
| F6527 | -41 | -18 | 63 |
| F6559 | -44 | -14 | 55 |
| F6839 | -37 | -20 | 60 |
| F7343 | -48 | -12 | 53 |
| F7370 | -46 | -7 | 53 |
| F7632 | -39 | -22 | 56 |
| F8082 | -37 | -19 | 67 |
| F8089 | -39 | -21 | 64 |
| F8311 | -38 | -14 | 69 |
| F8704 | -39 | -14 | 56 |
| F8859 | -42 | -15 | 56 |
| F9128 | -41 | -20 | 58 |
| F9138 | -37 | -15 | 57 |
| F9194 | -44 | -9 | 56 |
| F9563 | -42 | -14 | 65 |
| F9580 | -44 | -11 | 57 |
| F9611 | -35 | -19 | 66 |
| F9674 | -38 | -19 | 66 |
| F9788 | -47 | -9 | 51 |

**Figure S1.** Correlation matrix of the continuous independent variables


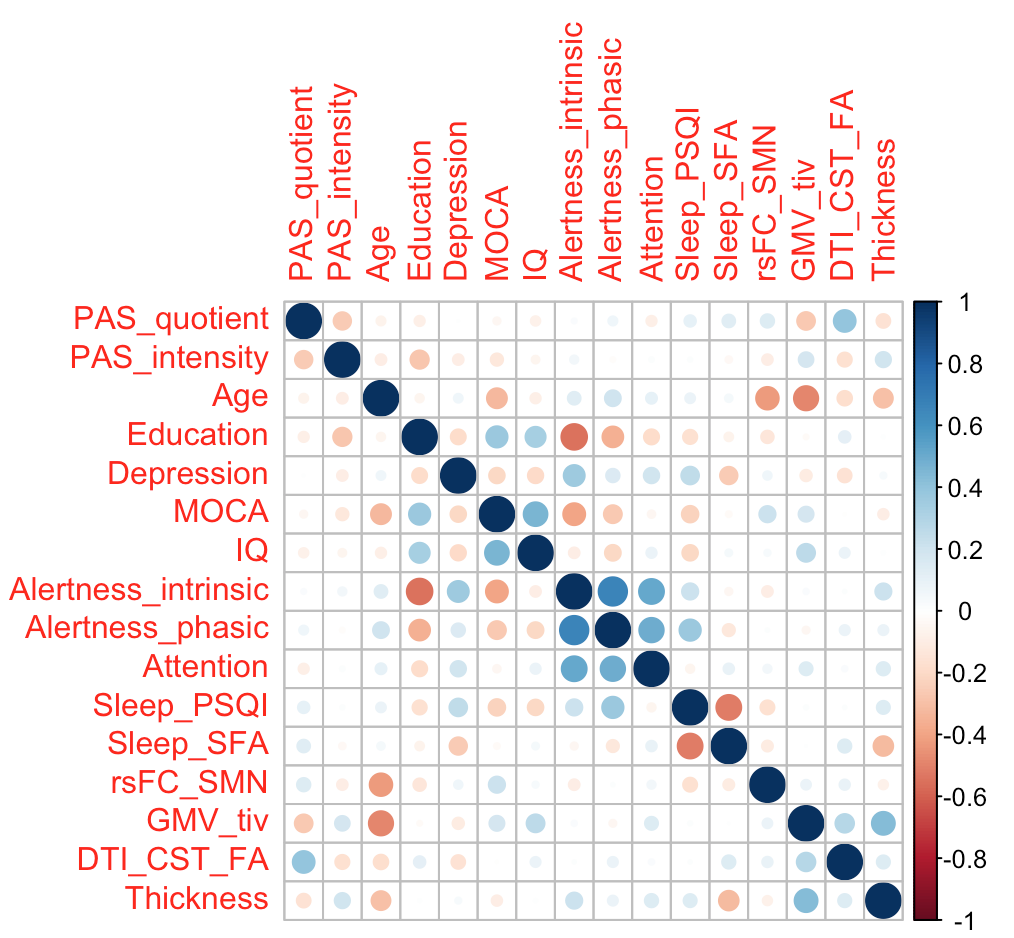

Supplement: Supplementary file 1 [file Data_Sheet_1.docx]
